# Exploring the Anti-Inflammatory Effect of Inulin by Integrating Transcriptomic and Proteomic Analyses in a Murine Macrophage Cell Model

**DOI:** 10.3390/nu15040859

**Published:** 2023-02-08

**Authors:** Federica Farabegoli, Francisco J. Santaclara, Daniel Costas, Mercedes Alonso, Ana G. Abril, Montserrat Espiñeira, Ignacio Ortea, Celina Costas

**Affiliations:** 1 ANFACO-CECOPESCA, 16, Crta. Colexio Universitario, 36310 Vigo, Spain; 2 Instituto de Investigaciones Marinas (IIM-CSIC), Eduardo Cabello 6, 36208 Pontevedra, Spain; 3 Department of Microbiology and Parasitology, Faculty of Pharmacy, University of Santiago de Compostela, 15898 Santiago de Compostela, Spain; 4 Proteomics Unit, Centro de Investigación en Nanomateriales y Nanotecnología (CINN-CSIC), Instituto de Investigación Sanitaria del Principado de Asturias (ISPA), 33011 Oviedo, Spain

**Keywords:** inulin, inflammation, bioactives, proteomics, transcriptomics

## Abstract

Inulin is a natural polysaccharide classified as a soluble fiber with demonstrated prebiotic activity. Prebiotics can reduce intestinal and systemic inflammation through modulation of the gut microflora and their metabolites. Additionally, extensive research is illuminating the role of macrophages in the interaction between gut microbiota and many systemic inflammatory diseases. In this study, the anti-inflammatory properties of inulin were evaluated using a murine macrophage cell model (RAW 264.7) of inflammation, and the immunomodulatory mechanism was investigated using omics technologies. The cells underwent comprehensive transcriptomic and proteomic analyses to identify the mechanisms responsible for the observed anti-inflammatory phenotype. Functional analyses of these omics results revealed two potential mechanisms that may lead to an overall reduction in cytokine and chemokine transcription: the inhibition of the NF-κB signaling pathway, leading to the downregulation of proinflammatory factors such as COX2, and the promotion of the phase II defense protein Hmox1 via the Nrf2 pathway. This study provides promising targets for research on immune modulation by dietary fibers and offers new strategies for the design of functional ingredients, foods, and nutraceutical products, which could ultimately lead to personalized nutrition and improved consumer health.

## 1. Introduction

Inulin is a fermentable dietary fiber recently approved by the Food and Drug Administration as a supplement to improve the nutritional value of food products [1]. It is a type of fructan polysaccharide primarily obtained from chicory root. The consumption of a fiber-rich diet has been linked to a reduced incidence of inflammatory and autoimmune diseases. Therefore, some in vivo studies in rats have been conducted to demonstrate that consuming inulin can suppress type-1 inflammatory responses in the gut [2]. Certain lymphocyte subsets play a role in regulating immune responsiveness, maintaining intestinal barrier function, and suppressing chronic inflammation [3]. Intestinal macrophages help maintain tissue homeostasis, especially in resolving inflammation [4]. Chronic inflammatory diseases of the gut, such as inflammatory bowel disease and celiac disease, occur due to breakdowns in immune regulatory networks in the intestine. A deeper understanding of the molecular pathways involved in suppressing inflammation could lead to new targets to promote remission or ameliorate of such chronic diseases [3,4]. Prebiotics, which have been shown to reduce the release of mucosal proinflammatory cytokines and increase the immunoregulatory transforming growth factor-β [5], a potent inhibitor of lymphocyte function with a key role in normal immune regulation [6], are promising candidates.

Recently, in vitro studies in murine macrophages (RAW 264.7) using transcriptomic analysis confirmed that inulin and inulin-type fructans can induce an anti-inflammatory phenotype by regulating gene expression and transcriptional pathways in macrophages exposed to lipopolysaccharides (LPS). These studies have produced contradictory results, with some showing little capacity for inulin to directly modulate LPS-induced inflammatory responses [2], while others have demonstrated strong anti-inflammatory effects against LPS-induced inflammation, potentially achieved by inhibiting the activation of proinflammatory signaling pathways promoted by nuclear factor (NF)-κB transcription factor [7]. NF-κB regulates the expression of proinflammatory cytokines, chemokines, adhesion molecules, and inducible enzymes (such as cycloxygenase-2 and inducible nitric oxide synthase), as well as specific proteins involved in the immune response (such as major histocompatibility complex and costimulatory molecules) and regulatory cytokines (such as interleukin (IL) 2, IL12, and interferon), which control lymphocyte proliferation and differentiation [8].

In recent years, with the goal of understanding the biological phenomena related to the intake of bioactive compounds and their regulatory mechanisms, omics approaches such as proteomics and transcriptomics have been increasingly used to study the bioactive role of foods [9]. These technologies allow for the analysis of thousands of genes or proteins per sample, providing comprehensive information on the bioactive mechanisms of action. Transcriptomics examines the complete set of transcripts in a cell and their quantity for a specific developmental stage or physiological condition, cataloging all types of transcripts (including mRNAs, non-coding RNAs, and small RNAs) with the aim of determining the transcriptional structure of genes, splicing patterns, and other post-transcriptional modifications and to quantify the changing expression levels of each transcript under different conditions [10]. Proteomics, on the other hand, is defined as the large-scale analysis of proteins in a particular biological system at a given time [11]. From a biological perspective, transcriptomic analysis provides information on the current state of gene expression, reflecting both transcriptional and post-transcriptional regulation. Proteomics, on the other hand, examines the direct functional performers (proteins) produced and released in organisms. Integrating omics data from proteomic and transcriptomic analysis can therefore enhance our understanding of the potentially complex mechanisms of biological phenotypes.

In this study, a culture system of LPS-activated murine macrophages was used to simulate inflammatory conditions, and two omics approaches (transcriptomics and proteomics) were applied to gain a deeper understanding of the effects of inulin on inflammation and its effects on inflammatory pathways.

## 2. Materials and Methods

### 2.1. Inuline Solutions

Inulin (Orafti^®^, ~88% purity) was supplied by BENEO GmbH (Mannheim, Germany). Inulin was diluted in MilliQ (Merck, Darmstadt, Germany) water (100 mg/mL) and stirred for 1 h at 25 °C. The solution was then sterilized through filtration (0.22 nm filter), and serial dilutions were made in sterilized MilliQ water to obtain the concentrations used in the cell-based assay (5 and 10 mg/mL).

### 2.2. Raw 267.4 Cell Culture

Cell cultures of the murine macrophage line RAW 264.7 (ATCC^®^ TIB71™, Manassas, VA, USA) were established using Dulbecco’s Modified Eagle’s Medium (DMEM, ATCC-30-2002, Thermo Fisher Scientific, Waltham, MA, USA) supplemented with 10% fetal bovine serum and 1% streptomycin–penicillin antibiotics. The cultures were maintained in a 37 °C incubator with 5% CO_2_ and a humid atmosphere. The RAW 264.7 cells were grown to approximately 80% confluence for subculture and were kept in fresh medium until the assay.

### 2.3. Anti-Inflammatory Assays in Raw 264.7

To assess the anti-inflammatory effects, cells were cultured as a monolayer in six-well polystyrene plates in an incubator at 37 °Cand 5% CO_2_. The experiment was conducted in sextuplicate and was initiated once the cells reached a confluence of approximately 99% in each well. Inflammation was induced in the cells by adding 1600 µL of *Escherichia coli* O111:B4 LPS diluted in the medium (100 ng/mL). After a 30 min incubation, 400 µL of inulin dilutions at 10 and 5 mg/mL were added to the experimental groups ‘LPS + I1’ and ‘LPS + I2’, respectively, followed by a 24 h incubation. Two control groups were also prepared as follows: the ‘LPS’ group was treated with LPS without anti-inflammatory agents, and the ‘LPS + H’ group was treated with LPS and 100 µM hydrocortisone. After 24 h, the medium was removed from the cell cultures and stored at −20 °C for analysis of nitrite and cytokine release. The concentrations of nitrites and cytokines in the cell supernatant were measured to determine which inulin treatment resulted in higher anti-inflammatory activity.

Once the supernatant was collected, the cell layers from each well were detached using a cell scraper in 0.5 mL of cell growth medium. The cell suspension from the LPS + I group with the higher anti-inflammatory activity was analyzed with transcriptomics and proteomics using the LPS group as a reference.

#### 2.3.1. Quantification of Nitric Oxide (NO)

Nitrite accumulation in the culture medium, which serves as an indicator of nitric oxide (NO) production, was quantified using an Invitrogen™ Griess reagent kit, following the manufacturer’s instructions. This method is based on the conversion of sulfanilic acid into a diazonium salt in acid solution through reaction with nitrite. The resulting diazonium salt is then coupled with N-(1-naphthyl)ethylenediamine to form an azo dye, which can be spectrophotometrically quantitated based on its absorbance at 548 nm.

#### 2.3.2. Quantification of Cytokines

The amounts of tumor necrosis factor-α (TNF-α) and interleukin-6 (IL-6) were determined by enzyme-linked immunosorbent assay (ELISA) kits, following the manufacturer’s instructions: a mouse TNF-α ELISA kit (ref BMS 607-3TWO) and an IL-6 mouse ELISA kit (ref KMC0062) supplied by Invitrogen™.

#### 2.3.3. Data Collection

For nitrites, IL-6, and TNF-α, the percentage of inhibition in the production of inflammatory markers by macrophages was calculated for each treatment group using the following equation:

$$\%Inhibition=\left(1-\frac{{\left[marker\right]}_{sample}}{{\left[marker\right]}_{control\;LPS}}\right)\times 100$$

### 2.4. Transcriptomic Analysis

#### 2.4.1. RNA Extraction, Library Preparation, and Sequencing

Three biological replicates were collected for each group (LPS + I1 and LPS) for RNA isolation. Cells were lysed, and RNA was isolated using an RNeasy Plus mini kit (QIAGEN) according to the manufacturer’s instructions for cell culture. The number of cells used per sample ranged from 1 × 10^6^ to 5·× 10^6^. RNA was quantitated using a Qubit 4 fluorometer (Thermo Fisher Scientific, Waltham, MA, USA) and a Qubit RNA HS assay kit, following the manufacturer’s instructions. The RNA integrity number (RIN) for each sample was measured with a TapeStation 4150 system (Agilent, Santa Clara, CA, USA) using the high-sensitivity RNA ScreenTape. The RIN values for all samples were between 7 and 9.5. From this total RNA, RNA sequencing (RNA-Seq) was performed for the transcriptomics assay, followed by quantification of the expression of different genes using qPCR.

#### 2.4.2. RNA-Seq Assay and Analysis

Targeted RNA sequencing for gene expression and transcriptome analysis was performed using and Ion AmpliSeq™ transcriptome mouse gene expression kit (Thermo Fisher Scientific, Waltham, MA, USA) on an Ion S5™ system (Thermo Fisher Scientific, Waltham, MA, USA) according to the manufacturer’s instructions. Briefly, this kit performs reverse transcription from previously extracted RNA and then amplifies the gene regions to evaluate their expression levels. A library was prepared for each sample for subsequent sequencing, and each sample library was labeled with a barcode sequence. Sequencing was performed with an Ion S5 instrument sequencer and an Ion 540 chip in two batches of eight samples each. Template preparation was performed on an Ion Chef instrument with and Ion 540™ Chef chemistry kit.

For each sequenced sample, a FASTQ file containing all the DNA sequences was obtained. The quality of the FASTQ files was checked using FastQC v.0.11.9 [12]. Raw reads were mapped to the GRCm39 (mm39) mouse reference transcriptome using Salmon v1.6.0 [13] in quasi-mapping mode to quantify transcript abundances. Additionally, the reads were aligned to the mouse reference genome using STAR aligner version 2.7.10a [14] to determine the genomic origin of the reads. The quality of the alignments was evaluated with Qualimap v2.2.1 [15], and MultiQC [16] was used to generate a single report summarizing all quality control results. The reference FASTA sequences and gene annotations necessary for the Salmon and STAR alignments were obtained from GENCODE [17] release M28 for the GRCm39 mouse genome assembly (https://www.gencodegenes.org/mouse/, accessed on 1 June 2021).

#### 2.4.3. Differential Expression Analysis

The DESeq2 R software package (version 3.14) [18] was used to perform differential expression analysis between the two groups, LPS + I1 vs. LPS. The transcript-level count abundances from Salmon were summarized at the gene level and imported to R using the tximport v1.22.0 package [19]. Gene symbols were linked to Ensembl (release 99) biotype annotations for the *Mus musculus* reference genome GRCm39 using the AnnotationHub R package (v.2.2.2) [20]. Differentially expressed genes (DEGs) were identified based on an absolute fold change (FC) value > 1.2 and a Benjamini–Hochberg adjusted *p*-value < 0.05. The ‘apeglm’ method was used for log2 FC shrinkage.

#### 2.4.4. Functional and Pathway Enrichment Analysis

The clusterProfiler R package [21] was used to perform gene ontology (GO) functional annotation and Kyoto Encyclopedia of Genes and Genomes (KEGG) pathway analysis on the DEGs. GO terms and KEGG pathways with adjusted *p*-values less than 0.05 were considered significantly enriched.

### 2.5. Proteomic Analysis

#### 2.5.1. Total Protein Extraction

Cells from experimental groups LPS + I1 and LPS were collected in Eppendorf tubes on ice and centrifuged at 4 °C for 2 min at 16,000× *g* to remove the supernatants. The pellets were washed twice with 1 mL of cold PBS and centrifuged under the same conditions. After the complete removal of the supernatant, the cells were lysed with 200 µL of lysis buffer (0.5% Triton X-100, 50 mM pH 7,4 Tris-HCl, and 150 mM NaCl). The samples were then centrifuged for 15 min, and the supernatants were transferred to new tubes. The total protein content of the supernatant was quantified using a Pierce™ 660 nm protein assay (Thermo Fisher Scientific, Waltham, MA, USA), and the samples were stored in a frozen state until future analysis.

#### 2.5.2. Sample Preparation for Liquid Chromatography–Mass Spectrometry (LC-MS) Analysis

Protein extracts were cleaned through cold acetone precipitation. The resulting protein pellets were resuspended in 0.2% Rapigest SF (Waters, Milford, USA), and their protein concentration was measured using a Qubit assay kit on a Qubit nanofluorimeter (Thermo Fisher Scientific, Waltham, MA, USA). For each sample, 30 μg of total protein content was subjected to liquid digestion with trypsin (Promega, Madison, WI, USA) at 37 °C in two steps (2 h and 15 h each, with a 1:40 trypsin-to-protein ratio) as previously described [22]. The peptide digests were diluted with 0.1% formic acid (FA) to 100 ng/μL of equivalent protein content and transferred to a nano-HPLC vial.

#### 2.5.3. diaPASEF LC-MS Analysis

Samples (2 μL, 200 ng of protein digest on column) were analyzed using a timsTOF Pro (Bruker, Billerica, MA, USA) Q-TOF mass spectrometer coupled with a nanoElute (Bruker, Billerica, MA, USA) LC system. The analysis was performed using a C18 Aurora series UHPLC emitter column (250 mm × 75 μm id, 1.6 μm, 120 Å pore size) (IonOpticks, Fitzroy, Australia) in a trap–elute configuration with an Acclaim PepMap C18 trap cartridge (5 mm, 300 μm id, 5 μm particle diameter, and 100 Å pore size) (Thermo Fisher Scientific). Peptides were eluted in a 45 min gradient from 5 to 30% B (5 to 25% B in 40 min, 25 to 30% B in 5 min), followed by 12 min of column cleaning at 80% B. The solvents used were water with 0.1% FA (A) ACN with 0.1% FA (B). The chromatography flow rate was 300 nL/min. As the peptides eluted from the chromatography, they were ionized in a captive nanoelectrospray source (Bruker, Billerica, MA, USA) at 1500 V. The mass spectrometer acquired samples using a data-independent acquisition parallel accumulation serial fragmentation (diaPASEF) method [23] consisting of 12 cycles that covered 34 mass-width windows (25 Da width, ranging from 350 to 1200 *m*/*z*), with two mobility windows each, resulting in 68 windows that covered the ion mobility range (1/K_0_) from 0.64 to 1.37 V s/cm^2^. These windows were optimized using the window editor utility in the instrument control software (timsControl, Bruker). Optimization was performed using one data-dependent acquisition (DDA) run that was previously acquired from a pool of samples. The utility loaded the run and represented its ion density in the *m*/*z* and ion mobility ranges (i.e., the mobility heatmap) to adjust the diaPASEF window coverage and ensure complete coverage. The collision energy was set as a function of ion mobility, following a straight line from 20 eV for 1/K_0_ of 0.6 V s/cm^2^ to 59 eV for 1/K_0_ of 1.6 V s/cm^2^. The TIMS elution voltage was calibrated linearly to obtain 1/K_0_ ratios using three ions from the ESI-L tuning mix (Agilent, Santa Clara, CA, USA) (*m*/*z* 622, 922, 1222) before each run, using the ‘Automatic calibration’ utility in the control software (timsControl, Bruker, Billerica, MA, USA).

#### 2.5.4. Data Analysis

To extract the quantitative data for peptides and proteins from the diaPASEF runs, the library-free workflow in DIA-NN [24] version 1.8 was used. This workflow consisted of two steps. First, the software built an in silico predicted library from a SWISS-PROT mouse (UP000000589) isoforms FASTA database (downloaded from www.uniprot.org, accessed on 9 July 2021) appended with common contaminant proteins, which contained a total of 25,483 sequence entries. The options ‘FASTA digest for library-free search/library generation’ and ‘Deep learning-based spectra, RTs and IMs prediction’ were enabled, missed cleavages were set to 0, the precursor change range was set to 2–4, and the precursor *m*/*z* range was set to 349–1500. In the second step, the generated predicted library was used to analyze the diaPASEF LC-MS runs with the following parameters: the neural network classifier was set to double-pass mode, the quantification strategy was set to ‘Robust LC (high precision)’, and the MBR option was enabled. The MS1 and MS2 accuracy and retention time window scans were set to 0 to allow DIA-NN to perform its automatic inference for the first run in the experiment. All other DIA-NN settings were left as default, using RT-dependent cross-run normalization and filtering the output at 1% FDR.

Fold-changes were calculated for the comparison of LPS + I1 vs. LPS. Differential expression analysis for each of the quantified proteins was performed using a Student’s *t*-test on log10-transformed data. *p*-values were adjusted for multiple hypothesis testing with Benjamini–Hochberg correction. Proteins that showed an adjusted *p*-value below 0.05 and an absolute FC above 1.2 were considered differentially expressed.

For functional analysis, iPathwayGuide version 2012 (Advaita Corporation, Ann Arbor, MI, USA) was used to analyze the significantly impacted pathways in the context of pathways obtained from the KEGG database (Release 96.0+/11–21 November 2020) and for GO analysis using gene ontologies from the GO Consortium database (October 2020).

### 2.6. Validation of Specific Genes by Quantitative Real-Time PCR

Total RNA previously obtained from the RAW 264.7 cell culture was treated with ezDNase and reverse-transcribed using SuperScrit IV VILO Master Mix (Invitrogen, Waltham, MA, USA). The concentration of cDNA was measured with a Qubit 4 fluorometer (Thermo Fisher Scientific, Billerica, USA) using a Qubit ssDNA assay kit following the recommendations of the manufacturer (Invitrogen, Waltham, MA, USA). The gene IDs of the genes quantified with quantitative polymerase chain reaction (qPCR) are Ptgs2 (PrimerBank ID 31981525a1), Oasl1 (PrimerBank ID 21630289a1), Fabp4 (PrimerBank ID 14149635a1), Slc37a2 (PrimerBank ID 225543197c2), Cp (PrimerBank ID 110347563c3), Lpl (PrimerBank ID 6678710a1), Il1a (PrimerBank ID 52669a1), Il1b (PrimerBank ID 6680415a1), Rgs16 (PrimerBank ID 190684664c3), Il6(PrimerBank ID 26354667a1), Il27(PrimerBank ID 21704110a1), and Hvcn1(PrimerBank ID 21311863a1). These primers were obtained from PrimerBank [25,26,27]. Additionally, eight housekeeping genes were tested (Hdgf, Alg2, Mlec, Hsp90b1, Brms1l, Rpl4, Gapdh, and Actb), with the first four obtained from the HRT Atlas Database [28] and the last four from Ruiz-Villalba et al. [29]. The amplification information for the qPRC assays is shown in Supplementary Table S1. The number of genes and the qPCR assay were configured following the recommendations of “The MIQE guidelines” [30]. Quantification was performed in triplicate using 20 μL reactions, each containing 35 ng of cDNA, 500 nM of each primer, and PowerUp™ SYBR™ Green Master Mix (Thermo Fisher Scientific, Billerica, MA, USA). The reactions were performed on a ViiA-7 real-time PCR system (Thermo Fisher Scientific, Billerica, MA, USA) with an initial step at 50 °C for 2 min to activate uracil-DNA glycosylase (UDG) to minimize PCR contamination and initial activation at 95 °C for 2 min, followed by a 3-step program of 40 cycles as follows: 95 °C for 15 s, 60 °C for 60 s, and 72 °C for 30 s. After completion of the PCR, a dissociation curve was generated, starting at 50 °C and increasing to 95 °C at a heating ramp of 0.05 °C per second. Each gene was analyzed on the same plate for all samples, so inter-run calibration was not necessary. The expression data were analyzed with qBase+ [31] after adjusting for amplification efficiency of each transcript.

## 3. Results

### 3.1. Anti-Inflammatory Assays in Raw 264.7

**Figure 1:**
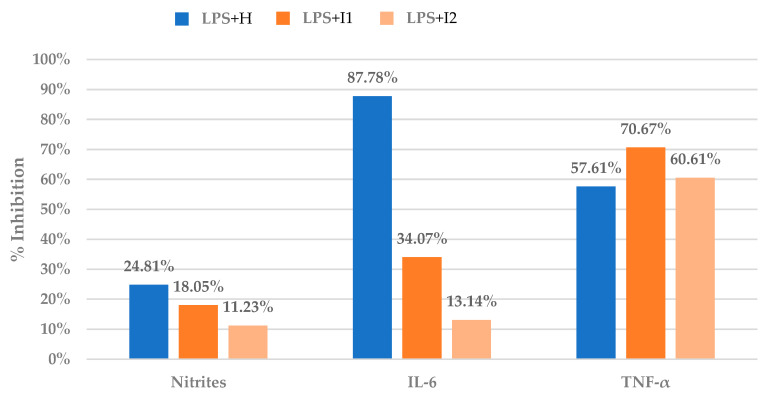
Results of anti-inflammatory assay on a macrophage cell line (RAW 264.7) of two treatment concentrations (LPS + I1 and LPS + I2; see Section 2.3). Results are calculated as percentage of inhibition of mediators’ release against sample LPS. The positive control is LPS + H.

The results of the anti-inflammatory assay of inulin on RAW 264.7 cells are presented as percentages of inhibition of LPS-induced inflammation based on the release of inflammatory mediators (nitrites, IL-6, and TNF-α). Figure 1 shows the inhibitory capacity of two concentrations of the treatment (LPS + I1 and LPS + I2) and the positive control (LPS + H) on the release of these mediators.

Figure 1 illustrates that inulin inhibits the release of the measured inflammatory mediators to a certain degree in a dose-dependent manner. The higher concentration of inulin used in the LPS + I1 treatment (10 mg/mL) resulted in greater inhibition of mediator release compared to the lower concentration of inulin used in LPS + I2 (5 mg/mL). Therefore, the higher concentration of inulin (I1) was chosen for further investigation of inulin’s anti-inflammatory activity in RAW 264.7 cells using a multiomics strategy. The inhibition of TNF-α release by both concentrations of inulin exceeded that of the anti-inflammatory drug hydrocortisone (positive response control). However, inulin’s inhibition of IL-6 release was only a fraction of that produced by hydrocortisone (34% vs. 88% inhibition, respectively), suggesting that TNF-α may play a key role in explaining inulin’s anti-inflammatory effects.

### 3.2. Transcriptomic Analysis

To examine the anti-inflammatory effect of inulin on the transcriptome of LPS-stimulated cells, RNA-Seq and differential expression analyses were performed on the LPS + I1 and LPS experimental groups. The normalized count data for all mapped genes are presented in Supplementary Table S2.

**Figure 2:**
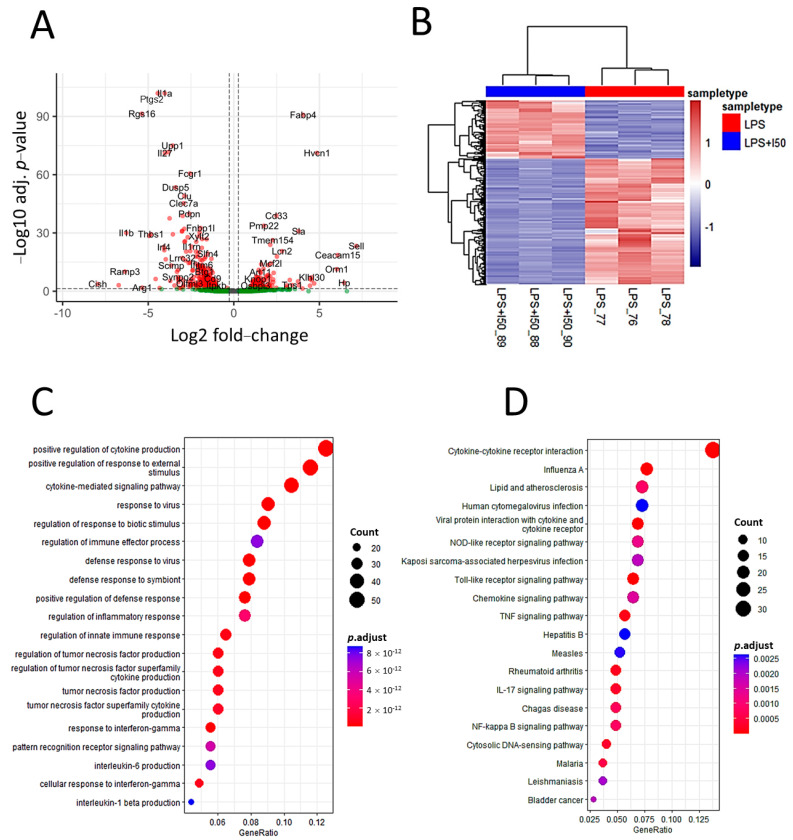
Transcriptomic analysis of inulin-treated, LPS-stimulated cells. (**A**) Volcano plot showing the relative abundances of transcripts. Transcripts with an FC value > 1.2 and an adjusted *p*-value < 0.05 were considered to be differentially expressed between the inulin treatment group and the LPS-stimulated group. (**B**) Hierarchical heatmap showing the expression patterns of LPS-stimulated cells treated with and without inulin using the differentially expressed genes. Samples are displayed in columns, and genes are presented in rows. (**C**) GO enrichment analysis showing top 20 enriched GO biological process terms. (**D**) Pathway enrichment analysis showing the top 20 enriched KEGG pathways.

Differential expression analysis using DESeq2 resulted in 447 differentially expressed genes (DEGs), with 306 genes downregulated and 141 genes upregulated (Supplementary Table S3). A volcano plot was created to display the distribution of these DEGs (Figure 2A). The heatmap of the DEGs showed a clear separation of the transcriptional signatures between the two groups and tight clustering among biological replicates (Figure 2B), indicating the differential expression pattern produced by the effect of inulin. Figure 2C,D show the top 20 significant biological processes (GO terms) and KEGG pathways, respectively, as the main results of the functional and pathway enrichment analysis.

**Figure 3:**
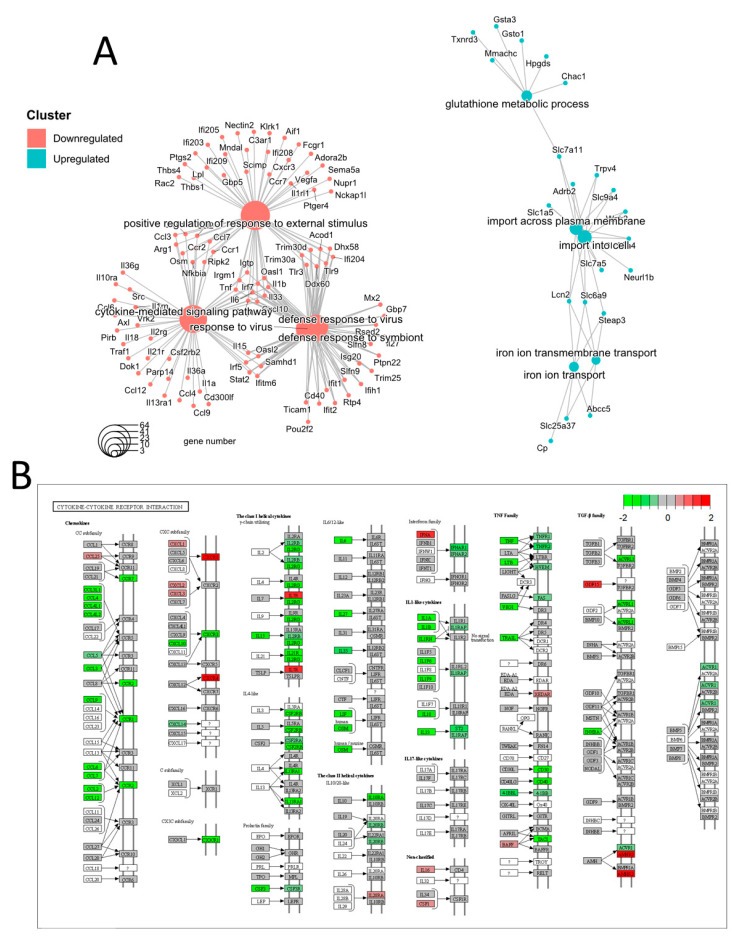
Transcriptomics showed an intense downregulation of cytokine production and regulation processes. (**A**) Category net plot of GO terms enriched for over− and underexpressed genes separately. (**B**) Cytokine–cytokine receptor interaction pathway showing integrated transcriptomics data. Genes overexpressed (in red) or underexpressed (green) according to the transcriptomic analysis are highlighted.

When over- and underexpressed genes were analyzed separately (Figure 3A), biological processes related to cytokine production and signaling, as well as response to viruses and biotic stimuli, stood out as the most downregulated clusters, accounting for a large proportion of the DEGs. In contrast, the upregulated processes accounted for fewer DEGs and were primarily related to transmembrane transport. Figure 3B shows the most overrepresented pathway, the cytokine–cytokine receptor interaction pathway, highlighting the DEGs that were mapped to this pathway as a result of the transcriptomic analysis.

### 3.3. Proteomic Analysis

The analysis of six biological replicates of the LPS + I1 and LPS groups resulted in the quantification of a total of 6839 unique proteins identified and quantified using only proteotypic (gene-specific) peptides, with a 1% FDR (Supplementary Table S4). The missing data comprised 4.2% (4644 data points out of a total of 109,424 measurements), demonstrating the high level of completeness achieved by the DIA-NN library-free workflow. To evaluate the quantitative precision of the workflow, the experimental coefficients of variation (CV) of the areas quantified for each unique protein were calculated for five technical replicates of one randomly selected sample. The average CV was 8.5%, with 6254 proteins (91.4%) with a CV below 20% and 4897 proteins (71.6%) with a CV value below 10%. For downstream differential expression and functional analysis, only proteins with a CV ≤ 20.0% and quantified in at least 50% of the samples in each group were considered. This resulted in a total of 6065 proteins meeting these criteria.

**Figure 4:**
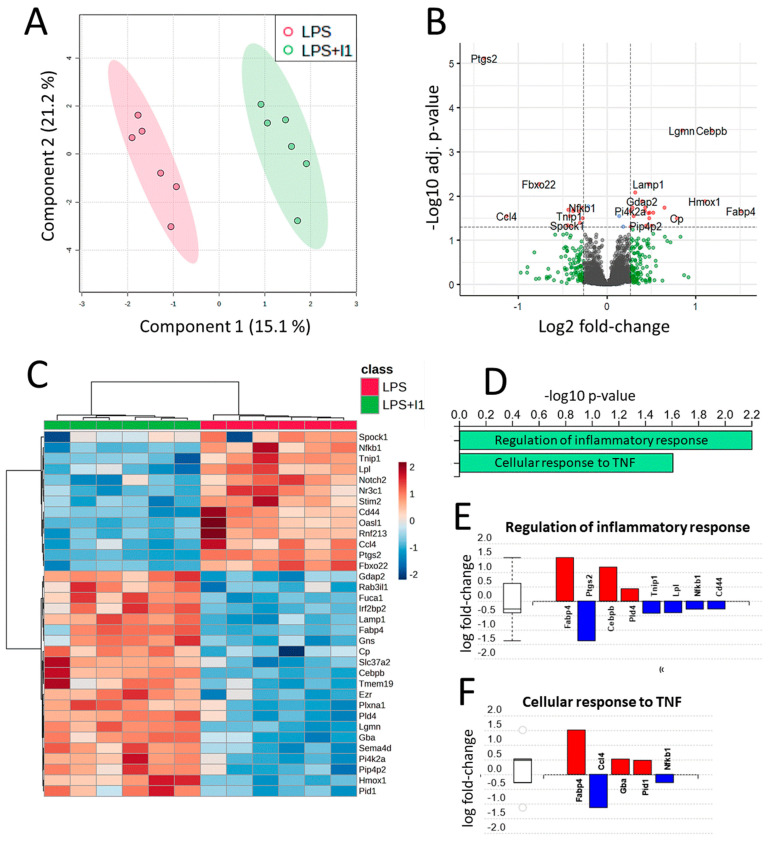
Overview of proteomic analysis results. (**A**) Partial least squares discriminant analysis (PLSDA) two-component score plot including all 6065 considered quantified proteins. Bioreplicates from both experimental groups are clearly separated. (**B**) Volcano plot showing proteins with differential expression in LPS-activated RAW 264.7 as a result of inulin treatment. Dotted lines show adjusted *p*-values < 0.05 and 1.2 fold-change cutoffs; proteins above both thresholds are shown in red. (**C**) Heatmap showing the unsupervised clustering of the 12 analyzed bioreplicates using the 34 proteins with differential expression as measured by diaPASEF LC-MS. Samples from the LPS + I1 and reference (LPS) groups are shown in green and red, respectively. (**D**) GO analysis showing significant (adjusted *p*-values < 0.05) biological processes. Gene perturbation bar plots for the affected proteins within biological processes, (**E**) regulation of inflammatory response, and (**F**) cellular response to tumor necrosis factor (TNF). Overexpressed proteins are shown in red, and under-expressed proteins are shown in blue.

The expression profiles of these proteins revealed a clear separation between the samples from the two experimental groups through partial least squares discriminant analysis (Figure 4A). The differential expression analysis of LPS + I1 vs. LPS (detailed results can be found in Supplementary Table S5) resulted in 34 differentially expressed proteins (DEPs) with adjusted *p*-values < 0.05 and FC values > 1.2 (Table 1). A total of 21 proteins showed increased expression following inulin treatment, while 13 proteins were downregulated (Figure 4B,C).

**Figure 5:**
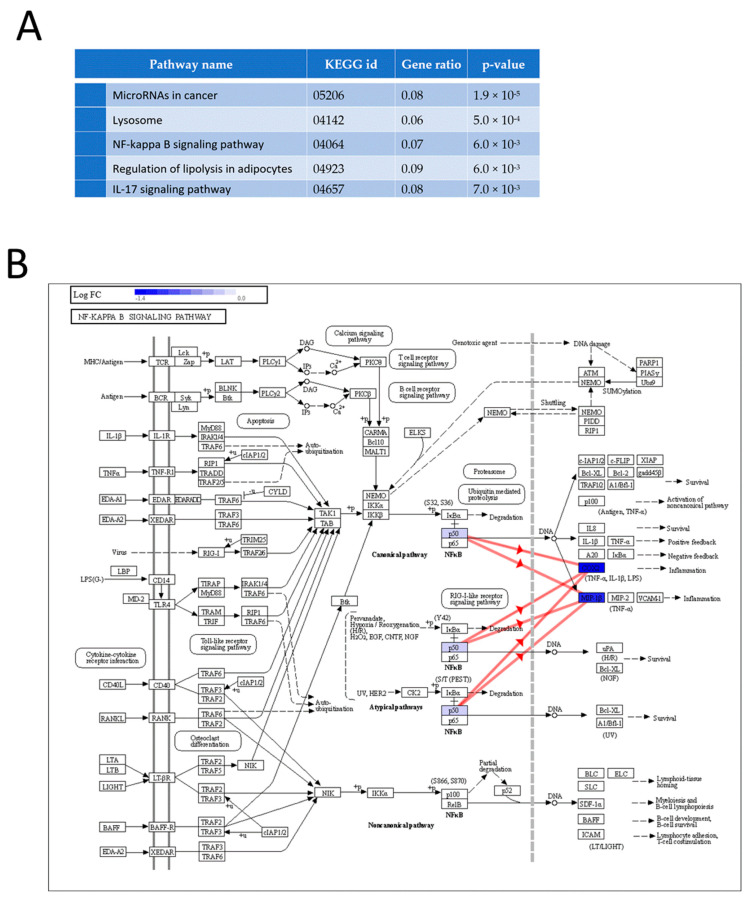
Proteomic pathway impact analysis. (**A**) Top five impacted pathways according to the proteomic analysis. (**B**) Effect of inulin on the NF-κB signaling pathway of LPS-activated RAW 264.7 cells, highlighting regulated proteins and showing coherent cascades.

Regarding the functional analysis of proteomics data, two GO terms were found to be statistically significant using iPathwayGuide’s ‘smallest common denominator’ algorithm (Figure 4D–F). To determine the biological pathways in which the 34 identified DEPs are involved, pathway impact analysis was performed using iPathwayGuide. This tool employs an impact analysis approach that takes into account not only the over-representation of DEPs in a given pathway (i.e., enrichment analysis) but also topological information, such as the direction and type of all signals in a pathway, as well as the position, function, and type of each protein [32]. The top five pathways are shown in Figure 5A,B, including the NF-κB signaling pathway, highlighting regulated proteins as identified by our proteomic analysis.

### 3.4. Validation of Selected Differentially Expressed Genes by qPCR

For validation of the transcriptomics and proteomics results by qPCR, two groups of genes were selected. The first group consisted of six genes that showed differentially expression at both the proteomic and transcriptomic levels, while the second group consisted of six genes from the RNA-Seq assay that presented high FC values, all of them showing statistically significant differential expression (adj. *p*-value < 0.05) (see Supplementary Table S1). One of the genes in the latter group, Hvcn1, was later excluded due to dimer formation during the PCR. The stability of the eight normalizer genes was evaluated using the geNorm tool [31,33] integrated in qBase. The results indicated that the MLEC and RPL4 genes were the most stable and were selected for normalization of relative quantities. The expression levels of the targeted genes were obtained for each sample, and the FC and significance levels were calculated for comparison between LPS + I1 and LPS. The results are presented in Table 2, along with the proteomics and transcriptomics data for the targeted genes.

It was observed that the expression levels of five out of the six genes evaluated by proteomics, RNA-Seq, and qPCR showed consistent changes (either up- or downregulated). Only one gene, Slc37a2, showed contradictory results, with a slight upregulation in proteomics but downregulation in both transcriptomics and qPCR measurements. Additionally, the six genes analyzed only by RNA-Seq and qPCR also showed consistent results. Thus, the results obtained by the three methodological approaches (proteomics, RNA-Seq, and qPCR) are highly concordant.

## 4. Discussion

The consumption of dietary fiber inulin can be considered a good strategy to reduce the incidence of inflammatory and autoimmune gut diseases, but its mechanism of action has not yet been clarified [2,7]. The integration of omics approaches in cellular and molecular biology studies is increasingly important in understanding biological phenomena, as the findings of single omics research strategies are often not comprehensive. For this reason, in this study, we coupled the application of transcriptomic and proteomic analysis to a well-characterized in vitro culture system of LPS-activated murine macrophages.

The analysis of the most downregulated genes derived from transcriptome analysis (Supplementary Table S3) revealed a predominant inhibition of many interleukins (e.g., IL-1α, IL-1β, IL-6, IL-15, IL-18, IL-27, IL-33, IL-36A, and IL-36G), interleukin and chemokine receptors and ligands, and other genes related to the defensive response, such as TNF and interferon regulators. Cytokines and chemokines regulate inflammatory processes by mediating immune cell recruitment and intracellular signaling mechanisms that characterize inflammation [34]. GO and KEGG enrichment analysis were performed on the transcriptomics data to globally explore the biological significance associated with the DEGs. GO functional annotation was explored at the subontology level. For biological processes, many of the most significant terms are related to cytokine- and interleukin-related processes, as well as defense and inflammatory responses, including the response to viral infections (Figure 2C).These processes, which were the most significant of the GO enrichment analysis, correspond to underexpressed genes, as derived by analyzing over- and underexpressed genes separately (Figure 3A), namely the cytokine-mediated signaling pathway, positive regulation of cytokine production, response to virus, regulation of response to biotic stimulus, and positive regulation of response to external stimulus. Regarding KEGG pathways, the enrichment analysis also indicated an impact on cytokine mechanisms, as DEGs were mainly involved in cytokine receptors and interaction with viral infections (Figure 2D). In fact, the top over-represented pathway derived from the transcriptomics analysis was the cytokine–cytokine receptor interaction pathway. Integrating the RNA expression data into this KEGG pathway once again shows that inulin has a general effect on this pathway by strongly suppressing the transcription of many chemokines and interleukins (Figure 3B), contributing to the inflammation suppression phenotype observed in the LPS-activated macrophages.

Transcriptomics, although not RNA-Seq, have previously been applied to study the anti-inflammatory effects of inulin in macrophages, but the results are inconclusive. On one hand, Myhill et al. claimed that inulin has little ability to directly modulate LPS-induced responses but that its anti-inflammatory effect may depend on the in vivo production of short-chain fatty acids by the gut microbiota. [2]. On the other hand, Meng et al. [7] observed a reduction in the expression of inflammatory factors (e.g., NF-κB, TNF-α, and IL-6) as a direct effect of inulin and suggested that the anti-inflammatory activity of inulin may be independent of gut microbiota and may be mediated by the inhibition of the NF-κB pathway. However, it is important to note that neither of these studies used RNA-Seq. The first study used a gene expression microarray kit and targeted approaches (ELISA and qPCR), while the second study used qPCR to target only four genes. In our study, we used RNA-Seq for transcriptomic analysis, thus providing a more comprehensive and in-depth insight into gene expression. RNA-Seq is a transcriptome profiling approach that uses deep-sequencing technology, providing a far more precise measurement of transcripts and their isoforms than other methods [10]. Our results from the transcriptomic analysis are in agreement with the hypothesis of Meng et al. [7], as we observed a general downregulation of cytokine signaling pathways and related defense mechanisms.

To investigate the mechanisms altered by inulin at the protein level, we used a massive quantitative proteomics approach. For the proteomic analysis, we employed diaPASEF LC-MS/MS acquisition, which combines some of the most significant recent developments in MS, including data-independent acquisition (DIA), ion mobility, and parallel accumulation serial fragmentation (PASEF). This methodology enables routine, reproducible, and highly efficient quantification of proteomes at a much greater depth [23].

The most significant protein in our proteomic analysis was prostaglandin G/H synthase 2 (Ptgs2) or cyclooxygenase 2 (COX-2), with an adjusted *p*-value of 7.63 × 10^−6^ and an FC of 0.38, which is 2.63 times less abundant in the LPS + I1 group. This finding was also supported by our transcriptomic analysis, which showed Ptgs to be strongly downregulated (log2FC = −4.44). Ptgs2 has been linked to inflammatory processes via the prostaglandin biosynthesis pathway. COX-2 converts arachidonic acid into prostaglandin H2, which is subsequently metabolized into potent proinflammatory signaling molecules that play crucial roles in diseases such as inflammation and cancer [35] (i.e., prostaglandins). Cyclooxygenase activity can be inhibited by non-steroidal anti-inflammatory drugs [36]. Our results suggest that inulin has a direct anti-inflammatory effect, as it strongly downregulates this key enzyme.

Another key protein that was significantly downregulated by the inulin treatment was 2′-5′ oligoadenylate synthetase-like 1 (Oasl1), with an FC of 0.74. The corresponding gene was also found to be strongly underexpressed in our transcriptomic assay (log2fC = −2.64), as validated by qPCR. Oasl1 is an interferon (IFN)-induced protein that mediates the antiviral action of type I IFN [37]. IFN, which plays a central role not only in viral infections but also in inflammatory-related mechanisms, is kept under close control to prevent unnecessary damage from hyperinflammatory responses [38]. Depending on the stimulus, Oasl can either promote or inhibit type I IFN, influencing the defensive behavior of the immune system [38,39]. Several studies have demonstrated that Oasl1 deficiency in mice enhances defense responses by positively regulating type I IFN [40,41]. Therefore, the role of Oasl1 in the anti-inflammatory effects of inulin is unclear.

Three proteins showed more than a two-fold significant increase as a result of inulin treatment: heme oxygenase 1 (Hmox1, FC = 2.14), CCAAT/enhancer-binding protein beta (Cebpb, FC = 2.27), and fatty-acid-binding protein 4 (Fabp4, FC = 2.85). Hmox1 is an anti-inflammatory effector regulated by the nuclear factor erythroid 2-related factor 2 (Nrf2) and expressed in response to diverse stimuli such as antioxidants, ROS, and heavy metals [42]. The Nrf2 pathway is a phase II enzyme induction system, where Hmox1 and other detoxifying/antioxidant proteins are induced by the binding of the transcription factor Nrf2 to the antioxidant response element (ARE) DNA region [43]. Several bioactive phytochemicals with anti-inflammatory and anticancer activities, such as curcumin and sulforaphane, have been shown to act through the Nrf2 transcriptional activation pathway [43]. The significant increase we found in the expression of Hmox1 could indicate that inulin activates the Nrf2 pathway, as do these phytochemicals. However, in our data, we did not find an upregulation of Nrf2, other phase II defense proteins, or cytoprotective proteins that have been described as Nrf2-induced. On the other hand, we did find a significant increase in Cebpb (FC = 2.27), a transcriptional regulator that binds in this Nrf2-ARE antioxidant defense pathway not to the ARE but to the XRE (xenobiotic response element) DNA region. Therefore, our data suggest that inulin has a direct effect on the Nrf2 pathway through activation of the XRE. Regarding Fabp4 (FC = 2.85), recent studies have shown that it is increased in chronic inflammatory diseases such as osteoarthritis and in cellular models of inflammation, which have shown that its knockdown can suppress inflammation and oxidative stress by inhibiting the NF-κB signaling pathway [44]. Fabp4 has been described as playing a role in activating innate immune responses by polarizing macrophages to proinflammatory subtypes [45]. Several in vitro and in vivo studies have indicated that Fabp4 may be a link between inflammation, obesity, and metabolic syndrome [46,47]. Its deficiency has been found to be protective against the development of insulin resistance and atherosclerosis and to reduce the expression of inflammatory cytokines in macrophages [48]. We also found a considerable increase in Fabp4 by transcriptomics (log2FC = 4.05), as validated by qPCR (log2FC = 3.76) (Table 1). It might therefore be expected that one of the inulin mechanisms for reducing inflammation could occur through inhibition of Fabp4. However, our results show Fabp4 overexpression in the LPS + I1 group, indicating that the direct anti-inflammatory effect of inulin is not produced by reducing the levels of this proinflammatory protein.

GO components and pathways affected by inulin treatment were analyzed using iPathwayGuide software. Only two GO terms for biological processes were found to be statistically significant when using iPathwayGuide’s ‘smallest common denominator’ algorithm: regulation of inflammatory response (GO: 0050727) and cellular response to TNF (GO: 0071356), with eight and five DEPs, respectively (Figure 4D–F). This result highlights differences in protein expression related to inflammation response processes. Among the proteins involved in regulation of inflammatory response, GO analysis identified Ptgs2, TNF-α-induced protein 3-interacting protein 1 (Tnip1), lipoprotein lipase (Lpl), NF-κB (Nfkb1), and CD44 antigen to be downregulated in the LPS inflammatory cell model as an effect of inulin. Conversely, Fabp4, Cebpb, and phospholipase D 4 (Pld4) were upregulated by inulin (Figure 4E). In addition, the algorithm also identified five DEPs involved in cellular response to TNF (GO:0071356). Two of these proteins are also involved in regulation of inflammatory response, namely Fabp4 and Nfkb1, as described above. Among proteins, in addition to Nfkb1, chemokine ligand 4 (Ccl4) was downregulated in LPS + I1 compared to LPS, while glucosidase beta (Gba), and the phosphotyrosine interaction domain containing 1 (Pid1) were upregulated in LPS + I1 compared to LPS (Figure 4F).

Micro-RNAs in cancer (*p*-value 1.9 × 10^−5^), lysosome (*p*-value 5.0 × 10^−4^), the NF-κB signaling pathway (*p*-value 6 × 10^−3^), regulation of lipolysis in adipocytes (*p*-value 6 × 10^−3^), and the interleukin 17 (IL-17) signaling pathway (*p*-value 7 × 10^−3^) were found to be significantly affected by inulin in our cell model (Figure 5A). The dysregulation of micro-RNAs (miRNAs) has been widely observed in different stages of cancer, but this mechanism could be a confounding factor in our study, as the RAW 264.7 cell model is derived from a mouse tumor induced by the Abelson murine leukemia virus. On the other hand, lysosome, the NF-κB signaling pathway, and the IL-17 signaling pathway are considered relevant in confirming the hypothesis that inulin has immunomodulatory properties. Lysosomes are involved in several processes in macrophages, including endocytosis, phagocytosis, and autophagy, which are essential to both innate and adaptive immunity [49]. Specifically, lysosomes are involved in the release of cytokines and chemokines that promote inflammation during the immune response [50]. Gao et al. found that intrinsic lysosome proteins such as Lamp1 and lysosomal hydrolases decreased in abundance after pathogen treatment of RAW 264.7 macrophages [49]. In our study, we found a significant increase in Lamp1 and several lysosomal hydrolases (Supplementary Figure S1), suggesting that inulin reverses, to some extent, the inflammatory mechanisms observed in lysosomes. The other pathways strongly related to inflammation that were found to be impacted by inulin according to our analysis were the NF-κB and the IL-17 signaling pathways. NF-κB comprises a family of ubiquitous eukaryotic transcription factors that regulates genes involved in immunity, inflammation, and cell survival. It mediates pleiotropic effects of both external and internal stimuli in cellular signaling cascades, and many chemopreventive phytochemicals that are derived from the diet have been shown to suppress constitutive NF-κB activation [43]. In the NF-κB canonical pathway activated by TNF-α, IL-1, or byproducts of bacterial (i.e., LPS) or viral infection, NF-κB promotes the expression of COX2 and MIP-1β. In our proteomics analysis, we observed downregulation of these three key proteins, COX2 (Ptgs2), MIP-1β (Ccl4, chemokine (c-C motif) ligand 4), and p50 (Nfkb1, nuclear factor of kappa light polypeptide gene enhancer in B cells 1), one of the NF-κB dimer components (Figure 5B). This pathway was also found to be significantly inhibited in our transcriptomics results, further demonstrating the strong correlation between the two approaches (Supplementary Figure S2).

Finally, the IL-17 signaling pathway is a subset of cytokines, with crucial roles in both acute and chronic inflammatory responses and important roles in protecting the host against extracellular pathogens and promoting inflammatory pathology in autoimmune disease. At the core of this IL-17 signaling pathway, similar to what was found in the NF-κB signaling pathway, we found a reduction in NF-κB protein levels as an effect of inulin treatment (Supplementary Figure S3).

## 5. Conclusions

The application of two advanced omics approaches has clarified and provided deeper insights into the molecular-level effects of inulin in regulating inflammatory activity, as suggested by earlier in vitro and in vivo studies. Taken together, our results indicate that inulin leads to a decrease in NF-κB levels, which in turn downregulates the expression of inflammatory factors such as COX2 and therefore reduces proinflammatory prostaglandins. Furthermore, inulin also appears to stimulate the transcription of the phase II defense protein Hmox1 through the Nrf2 pathway. This effect of inulin on these pathways likely contributes to the overall reduction in cytokine and chemokine transcription observed in our transcriptomic study, ultimately resulting in an anti-inflammatory effect.

The study of bioactive molecules and their impact on the human body is a complex and constantly evolving field. Thus, further research on inulin should examine its interaction with the intestinal microbiome and other compounds present in food, using more advanced biological models. It would also be valuable to delve deeper into the anti-inflammatory properties of inulin at a local level within the intestinal epithelium. The availability of large-scale scientific data obtained through the use of advanced analytical techniques and increasingly realistic and reliable biological models can lead to a better understanding and enable the design of personalized nutrition.

## Figures and Tables

**Table 1:** Gene names for the differentially expressed proteins (adjusted *p*-value < 0.05 and fold change > 1.2) ranked by adjusted *p*-value. BH, Benjamini–Hochberg.

Genes	Fold Change	*p*-Value	BH Adj. *p*-Value
Ptgs2	0.38	1.26 × 10^−9^	7.63 × 10^−6^
Lgmn	1.80	1.61 × 10^−7^	0.000325
Cebpb	2.27	1.54 × 10^−7^	0.000325
Lamp1	1.39	4.45 × 10^−6^	0.005396
Fbxo22	0.59	4.09 × 10^−6^	0.005396
Fuca1	1.25	8.25 × 10^−6^	0.008340
Gdap2	1.31	1.56 × 10^−5^	0.013015
Hmox1	2.14	1.72 × 10^−5^	0.013015
Nfkb1	0.82	3.76 × 10^−5^	0.018145
Sema4d	1.57	3.39 × 10^−5^	0.018145
Irf2bp2	1.35	3.18 × 10^−5^	0.018145
Ezr	1.22	3.89 × 10^−5^	0.018145
Nr3c1	0.77	4.62 × 10^−5^	0.020000
Oasl1	0.74	5.10 × 10^−5^	0.020618
Rnf213	0.77	6.01 × 10^−5^	0.021778
Pi4k2a	1.20	6.10 × 10^−5^	0.021778
Pld4	1.34	6.82 × 10^−5^	0.021780
Fabp4	2.85	6.81 × 10^−5^	0.021780
Stim2	0.80	7.42 × 10^−5^	0.022509
Gba	1.43	8.42 × 10^−5^	0.023808
Gns	1.40	8.64 × 10^−5^	0.023808
Plxna1	1.38	9.19 × 10^−5^	0.024230
Slc37a2	1.23	1.27 × 10^−4^	0.028491
Tnip1	0.75	1.26 × 10^−4^	0.028491
Ccl4	0.46	1.19 × 10^−4^	0.028491
Cp	1.73	1.5 × 10^−4^	0.031392
Cd44	0.83	1.49 × 10^−4^	0.031392
Pid1	1.39	1.56 × 10^−4^	0.031473
Notch2	0.81	2.06 × 10^−4^	0.040399
Spock1	0.74	2.44 × 10^−4^	0.046330
Pip4p2	1.36	2.64 × 10^−4^	0.047034
Tmem192	1.42	2.71 × 10^−4^	0.047034
Lpl	0.76	2.63 × 10^−4^	0.047034
Rab3il1	1.21	2.89 × 10^−4^	0.048628

**Table 2:** Differential abundance for the proteins/genes subjected to validation by qPCR. Fold changes (LPS + I1 vs. LPS) and statistical significance are shown for proteomics, transcriptomics, and qPCR analyses. n.d., no data; ↑, up-regulated; ↓, down-regulated.

Protein/Gene Id	Name	Proteomics			Transcriptomics			qPCR		
		log2 FC	Adj. *p*-Value	Significant Change	log2 FC	Adj. *p*-Value	Significant Change	log2 FC	Adj. *p*-Value	Significant Change
Ptgs2	Prostaglandin-endoperoxide synthase 2	−1.38	7.6 × 10^−6^-	↓	−4.44	1.2 × 10^−102^	↓	−0.05	1.4 × 10^−3^	↓
Oasl1	2′-5′ oligoadenylate synthetase-like 1	−0.43	2.1 × 10^−2^	↓	−2.64	3.1 × 10^−15^	↓	−0.16	2.2 × 10^−3^	↓
Fabp4	Fatty acid-binding protein 4	1.51	2.2 × 10^−2^	↑	4.05	1.7 × 10^−91^	↑	3.76	1.4 × 10^−3^	↑
Slc37a2	Solute carrier family 37	0.30	2.8 × 10^−2^	↑	−1.16	4.2 × 10^−4^	↓	−0.84	1.5 × 10^−2^	↓
Cp	Ceruloplasmin	0.79	3.1 × 10^−2^	↑	0.13	5.4 × 10^−8^	↑	0.59	1.0 × 10^−2^	↑
Lpl	Lipoprotein lipase	−0.40	4.7 × 10^−2^	↓	−2.19	3.4 × 10^−26^	↓	−0.44	1.4 × 10^−3^	↓
Il1a	Interleukin 1 alpha	n.d.	n.d.		−4.03	1.2 × 10^−102^	↓	−0.11	1.3 × 10^−3^	↓
Il1b	Interleukin 1 beta	n.d.	n.d.		−6.28	9.4 × 10^−31^	↓	−0.01	6.0 × 10^−4^	↓
Rgs16	Regulator of G-protein signaling 16	n.d.	n.d.		−5.37	4.4 × 10^−92^	↓	−0.04	1.4 × 10^−3^	↓
Il6	Interleukin 6	n.d.	n.d.		−3.71	4.9 × 10^−6^	↓	−0.15	4.0 × 10^−3^	↓
Il27	Interleukin 27	n.d.	n.d.		−4.02	8.1 × 10^−72^	↓	−0.19	1.1 × 10^−3^	↓
Hvcn1	Hydrogen voltage-gated channel 1	n.d.	n.d.		4.86	6.6 × 10^−72^	↑	n.d.	n.d.	

## Data Availability

The mass spectrometry proteomics data were deposited with the ProteomeXchange Consortium via the PRIDE partner repository with the dataset identifier PXD032759.

## Supplementary Materials

The following supporting information can be downloaded at: https://www.mdpi.com/article/10.3390/nu15040859/s1. Supplementary Table S1. Amplification system for the genes of interest and for the genes used for normalization in the qPCR validation. Supplementary Table S2. Normalized counts of all mapped genes from the RNA-Seq assay. Supplementary Table S3. Differentially expressed genes according to the differential expression analysis with DESeq2. Supplementary Table S4. diaPASEF-based protein quantification data for all the samples. A total of 6839 proteins were quantified by diaPASEF LC-MS/MS after processing of the runs as described in the Section 2 and Section 2.5.4. Supplementary Table S5. Differential abundance test for comparison of the LPS + I1 vs. LPS groups. Proteins with CV ≤ 20.0% and quantified in at least 50% of the samples of each group were considered. Fold changes, resulting *p*-values, and Benjamini–Hochberg corrected *p*-values for each of the 6065 considered proteins are shown. Supplementary Figure S1. Effect of inulin on the lysosome pathway (KEGG: 04142) from RAW 264.7 LPS-induced inflammation model cells. Pathway diagrams overlayed with the measured protein fold change showing coherent cascades. Differentially expressed genes are represented with positive values in red. Supplementary Figure S2. Effect of inulin on the NF-κB signaling pathway showing integrated transcriptomics data. Genes overexpressed (in red) or underexpressed (green) according to the transcriptomic analysis are highlighted. Supplementary Figure S3. Effect of Inulin on the IL-17 signaling pathway (KEGG: 04657) from RAW 264.7 LPS-induced inflammation model cells. Pathway diagram, overlayed with the measured protein perturbation showing coherent cascades. Differentially expressed genes are represented with negative fold-change values in blue and positive values in red.

## References

[1] Gupta N., Jangid A.K., Pooja D., Kulhari H. (2019). Inulin: A Novel and Stretchy Polysaccharide Tool for Biomedical and Nutritional Applications. Int. J. Biol. Macromol..

[2] Myhill L.J., Jensen P., Zakeri A., Nielsen L.F., Jakobsen S.R., Mejer H., Thamsborg S.M., Nejsum P., Williams A.R. (2020). Effects of the Dietary Fibre Inulin and Trichuris Suis Products on Inflammatory Responses in Lipopolysaccharide-Stimulated Macrophages. Mol. Immunol..

[3] Izcue A., Coombes J.L., Powrie F. (2009). Regulatory Lymphocytes and Intestinal Inflammation. Annu. Rev. Immunol..

[4] Na Y.R., Stakenborg M., Seok S.H., Matteoli G. (2019). Macrophages in Intestinal Inflammation and Resolution: A Potential Therapeutic Target in IBD. Nat. Rev. Gastroenterol. Hepatol..

[5] Hoentjen F., Welling G.W., Harmsen H.J.M., Zhang X., Snart J., Tannock G.W., Lien K., Churchill T.A., Lupicki M., Dieleman L.A. (2005). Reduction of Colitis by Prebiotics in HLA-B27 Transgenic Rats Is Associated with Microflora Changes and Immunomodulation. Inflamm. Bowel Dis..

[6] Kehrl J.H. (1991). Transforming Growth Factor-β: An Important Mediator of Immunoregulation. Int. J. Cell Cloning.

[7] Meng Y., Xu Y., Chang C., Qiu Z., Hu J., Wu Y., Zhang B., Zheng G. (2020). Extraction, Characterization and Anti-Inflammatory Activities of an Inulin-Type Fructan from Codonopsis Pilosula. Int. J. Biol. Macromol..

[8] Moynagh P.N. (2005). The NF-ΚB Pathway. J. Cell Sci..

[9] Ortea I. (2022). Foodomics in Health: Advanced Techniques for Studying the Bioactive Role of Foods. TrAC Trends Anal. Chem..

[10] Wang Z., Gerstein M., Snyder M. (2009). RNA-Seq: A Revolutionary Tool for Transcriptomics. Nat. Rev. Genet..

[11] Pandey A., Mann M. (2000). Proteomics to Study Genes and Genomes. Nature.

[12] Andrews S. FastQC: A Quality Control Tool for High Throughput Sequence Data. Version 0.11.9. http://www.bioinformatics.babraham.ac.uk/projects/fastqc.

[13] Patro R., Duggal G., Love M.I., Irizarry R.A., Kingsford C. (2017). Salmon Provides Fast and Bias-Aware Quantification of Transcript Expression. Nat. Methods.

[14] Dobin A., Davis C.A., Schlesinger F., Drenkow J., Zaleski C., Jha S., Batut P., Chaisson M., Gingeras T.R. (2013). STAR: Ultrafast Universal RNA-Seq Aligner. Bioinformatics.

[15] Okonechnikov K., Conesa A., García-Alcalde F. (2016). Qualimap 2: Advanced Multi-Sample Quality Control for High-Throughput Sequencing Data. Bioinformatics.

[16] Ewels P., Magnusson M., Lundin S., Käller M. (2016). MultiQC: Summarize Analysis Results for Multiple Tools and Samples in a Single Report. Bioinformatics.

[17] Frankish A., Diekhans M., Ferreira A.-M., Johnson R., Jungreis I., Loveland J., Mudge J.M., Sisu C., Wright J., Armstrong J. (2019). GENCODE Reference Annotation for the Human and Mouse Genomes. Nucleic Acids Res..

[18] Love M.I., Huber W., Anders S. (2014). Moderated Estimation of Fold Change and Dispersion for RNA-Seq Data with DESeq2. Genome Biol..

[19] Soneson C., Love M.I., Robinson M.D. (2016). Differential Analyses for RNA-Seq: Transcript-Level Estimates Improve Gene-Level Inferences [Version 2; Peer Review: 2 Approved]. F1000Research.

[20] Morgan M., Shepherd L. AnnotationHub: Client to Access AnnotationHub Resources. R Package Version 2.2.2.

[21] Wu T., Hu E., Xu S., Chen M., Guo P., Dai Z., Feng T., Zhou L., Tang W., Zhan L. (2021). ClusterProfiler 4.0: A Universal Enrichment Tool for Interpreting Omics Data. Innovation.

[22] Ortea I., Gonzalez-Fernandez M.J., Ramos-Bueno R.P., Guil Guerrero J.L. (2018). Proteomics Study Reveals That Docosahexaenoic and Arachidonic Acids Exert Different in vitro Anticancer Activities in Colorectal Cancer Cells. J. Agric. Food Chem..

[23] Meier F., Brunner A.-D., Frank M., Ha A., Bludau I., Voytik E., Kaspar-Schoenefeld S., Lubeck M., Raether O., Bache N. (2020). DiaPASEF: Parallel Accumulation–Serial Fragmentation Combined with Data-Independent Acquisition. Nat. Methods.

[24] Demichev V., Messner C.B., Vernardis S.I., Lilley K.S., Ralser M. (2020). DIA-NN: Neural Networks and Interference Correction Enable Deep Proteome Coverage in High Throughput. Nat. Methods.

[25] Spandidos A., Wang X., Wang H., Seed B. (2010). PrimerBank: A Resource of Human and Mouse PCR Primer Pairs for Gene Expression Detection and Quantification. Nucleic Acids Res..

[26] Spandidos A., Wang X., Wang H., Dragnev S., Thurber T., Seed B. (2008). A Comprehensive Collection of Experimentally Validated Primers for Polymerase Chain Reaction Quantitation of Murine Transcript Abundance. BMC Genom..

[27] Wang X., Seed B. (2003). A PCR Primer Bank for Quantitative Gene Expression Analysis. Nucleic Acids Res..

[28] Hounkpe B.W., Chenou F., de Lima F., De Paula E.V. (2021). HRT Atlas v1.0 Database: Redefining Human and Mouse Housekeeping Genes and Candidate Reference Transcripts by Mining Massive RNA-Seq Datasets. Nucleic Acids Res..

[29] Ruiz-Villalba A., Mattiotti A., Gunst Q.D., Cano-Ballesteros S., Van Den Hoff M.J., Ruijter J.M. (2017). Reference Genes for Gene Expression Studies in the Mouse Heart. Sci. Rep..

[30] Bustin S.A., Benes V., Garson J.A., Hellemans J., Huggett J., Kubista M., Mueller R., Nolan T., Pfaffl M.W., Shipley G.L. (2009). The MIQE Guidelines: Minimum Information for Publication of Quantitative Real-Time PCR Experiments. Clin. Chem..

[31] Hellemans J., Mortier G., De Paepe A., Speleman F., Vandesompele J. (2007). QBase Relative Quantification Framework and Software for Management and Automated Analysis of Real-Time Quantitative PCR Data. Genome Biol..

[32] Tarca A.L., Draghici S., Khatri P., Hassan S.S., Mittal P., Kim J.-S., Kim C.J., Kusanovic J.P., Romero R. (2009). A Novel Signaling Pathway Impact Analysis. Bioinformatics.

[33] Vandesompele J., De Preter K., Pattyn F., Poppe B., Van Roy N., De Paepe A., Speleman F. (2002). Accurate Normalization of Real-Time Quantitative RT-PCR Data by Geometric Averaging of Multiple Internal Control Genes. Genome Biol..

[34] Turner M.D., Nedjai B., Hurst T., Pennington D.J. (2014). Cytokines and Chemokines: At the Crossroads of Cell Signalling and Inflammatory Disease. Biochim. Biophys. Acta—Mol. Cell Res..

[35] Orlando B.J., Malkowski M.G. (2016). Substrate-Selective Inhibition of Cyclooxygeanse-2 by Fenamic Acid Derivatives Is Dependent on Peroxide Tone. J. Biol. Chem..

[36] Blobaum A.L., Marnett L.J. (2007). Structural and Functional Basis of Cyclooxygenase Inhibition. J. Med. Chem..

[37] Kakuta S., Shibata S., Iwakura Y. (2002). Genomic Structure of the Mouse 2′,5′-Oligoadenylate Synthetase Gene Family. J. Interf. Cytokine Res..

[38] Lee M.S., Kim B., Oh G.T., Kim Y.-J. (2013). OASL1 Inhibits Translation of the Type I Interferon–Regulating Transcription Factor IRF7. Nat. Immunol..

[39] Chang Y., Kang J.-S., Jung K., Chung D.H., Ha S.-J., Kim Y.-J., Kim H.Y. (2022). OASL1-Mediated Inhibition of Type I IFN Reduces Influenza A Infection-Induced Airway Inflammation by Regulating ILC2s. Allergy Asthma Immunol. Res..

[40] Sim C.K., Cho Y.S., Kim B.S., Baek I.-J., Kim Y.-J., Lee M.S. (2016). 2′–5′ Oligoadenylate Synthetase-like 1 (OASL1) Deficiency in Mice Promotes an Effective Anti-Tumor Immune Response by Enhancing the Production of Type I Interferons. Cancer Immunol. Immunother..

[41] Oh J.E., Lee M.S., Kim Y.-J., Lee H.K. (2016). OASL1 Deficiency Promotes Antiviral Protection against Genital Herpes Simplex Virus Type 2 Infection by Enhancing Type I Interferon Production. Sci. Rep..

[42] Ryter S.W. (2022). Heme Oxygenase-1: An Anti-Inflammatory Effector in Cardiovascular, Lung, and Related Metabolic Disorders. Antioxidants.

[43] Surh Y.-J. (2003). Cancer Chemoprevention with Dietary Phytochemicals. Nat. Rev. Cancer.

[44] Mao H., Han B., Li H., Tao Y., Wu W. (2021). FABP4 Knockdown Suppresses Inflammation, Apoptosis and Extracellular Matrix Degradation in IL-1β-Induced Chondrocytes by Activating PPARγ to Regulate the NF-ΚB Signaling Pathway. Mol. Med. Rep..

[45] Xiao Y., Shu L., Wu X., Liu Y., Cheong L.Y., Liao B., Xiao X., Hoo R.L.C., Zhou Z., Xu A. (2021). Fatty Acid Binding Protein 4 Promotes Autoimmune Diabetes by Recruitment and Activation of Pancreatic Islet Macrophages. JCI Insight.

[46] Makowski L., Boord J.B., Maeda K., Babaev V.R., Uysal K.T., Morgan M.A., Parker R.A., Suttles J., Fazio S., Hotamisligil G.S. (2001). Lack of Macrophage Fatty-Acid–Binding Protein AP2 Protects Mice Deficient in Apolipoprotein E against Atherosclerosis. Nat. Med..

[47] Boord J.B., Maeda K., Makowski L., Babaev V.R., Fazio S., Linton M.F., Hotamisligil G.S. (2004). Combined Adipocyte-Macrophage Fatty Acid–Binding Protein Deficiency Improves Metabolism, Atherosclerosis, and Survival in Apolipoprotein E–Deficient Mice. Circulation.

[48] Furuhashi M., Fucho R., Görgün C.Z., Tuncman G., Cao H., Hotamisligil G.S. (2008). Adipocyte/Macrophage Fatty Acid–Binding Proteins Contribute to Metabolic Deterioration through Actions in Both Macrophages and Adipocytes in Mice. J. Clin. Investig..

[49] Gao Y., Chen Y., Zhan S., Zhang W., Xiong F., Ge W. (2017). Comprehensive Proteome Analysis of Lysosomes Reveals the Diverse Function of Macrophages in Immune Responses. Oncotarget.

[50] Stow J.L., Ching Low P., Offenhäuser C., Sangermani D. (2009). Cytokine Secretion in Macrophages and Other Cells: Pathways and Mediators. Immunobiology.

