# Supplementary material for: Exploring the Anti-Inflammatory Effect of Inulin by Integrating Transcriptomic and Proteomic Analyses in a Murine Macrophage Cell Model
Source: Nutrients. 2023 Feb 8;15(4):859. doi: 10.3390/nu15040859 (PMC9965215; doi:10.3390/nu15040859)
Supplement: Supplementary file 1 [file nutrients-15-00859-s001.zip › nutrients-2160206-supplementary/Supplementary Materials_R1.pdf]

Supplementary Materials

Supplementary Table S1. Amplification system for the genes of interest and for the genes used in normalization in the qPCR validation.

	Gene ID	GenBank accession	PrimerBank ID	PrimerBank Validation		Primer Name	Forward Primer	Primer Name	Reverse Primer	Amplicon size	Tm
1	Ptgs2	NM_011198	31981525a1	yes	PROTEOMICS AND TRANSCRIPTOMICS COMMON GENES	Ptgs2_F	TGAGCAACTATTCCAAACCAGC	Ptgs2_R	GCACGTAGTCTTCGATCACTATC	74	60,3°C/62,9°C
2	Oasl1	NM_145209	21630289a1	yes		Oasl1_F	CAGGAGCTGTACGGCTTCC	Oasl1_R	CCTACCTTGAGTACCTTGAGCAC	191	61,6°C/64,6°C
3	Fabp4	NM_024406	14149635a1	yes		Fabp4_F	AAGGTGAAGAGCATCATAACCCT	Fabp4_R	TCACGCCTTTCATAACACATTCC	133	61,1°C/61,1°C
4	Slc37a2	NM_020258	225543197c2	no		Slc37a2_F	GGAGCTGTGGACAATGCCTT	Slc37a2_R	CCGAGAGGTAGTAACGCAGG	97	60,5°C/62,5°C
5	Cp	NM_007752	110347563c3	no		Cp_F	ATTTTCAACGGGCTGATGACA	Cp_R	GGAGTGGTAAATCCTGGTCACA	119	57,4°C/62,1°C
6	Lpl	NM_008509	6678710a1	yes		Lpl_F	GGGAGTTTGGCTCCAGAGTTT	Lpl_R	TGTGTCTTCAGGGGTCCTTAG	115	61,3°C/61,3°C
7	Il1a	NM_010554	52669a1	yes	RELEVANT DEGS	Il1a_F	CGAAGACTACAGTTCTGCCATT	Il1a_R	GACGTTTCAGAGGTTCTCAGAG	126	60,3°C/62,1°C
8	Il1b	NM_008361	6680415a1	yes		Il1b_F	GCAACTGTTCCTGAACTCAACT	Il1b_R	ATCTTTTGGGGTCCGTCAACT	89	60,3°C/59,4°C
9	Rgs16	NM_011267	190684664c3	no		Rgs16_F	CCATGCCTTCCTAAAGACGGA	Rgs16_R	GTACTCGTCAAAGATGTGGTGAG	127	61,3°C/62,9°C
10	Il6	AK089780	26354667a1	yes		Il6_F	CCAAGAGGTGAGTGCTTCCC	Il6_R	CTGTTGTTCAGACTCTCTCCCT	118	62,5°C/62,1°C
11	Il27	NM_145636	21704110a1	yes		Il27_F	CTGTTGCTGCTACCCTTGCTT	Il27_R	CACTCCTGGCAATCGAGATTC	177	61,3°C/61,3°C
12	Hvcn1	NM_001042489	21311863a1	yes		Hvcn1_F	ATGACTTCCCATGACCCAAAGG	Hvcn1_R	TCGTCCCCAACCACCGTAA	101	62,1°C/59,5°C

	Gene	Transcript ID	Primer Name	Forward	Tm Forward	Primer Name	Reverse	Tm Reverse	Amplicon bp	Efficiency	R2
13	**Hdgf**	ENSMUST00000005017	**Hdgf_F**	CATGAGAGCCTGTAGCCAC	60,3°C<br>59,5°C (salt)	**Hdgf_R**	GTGGGCTTAGAGGAGAGAG	58,6°C<br>59,5°C (salt)	159	100,2	0,98
14	**Alg2**	ENSMUST00000044148	**Alg2_F**	AACCACTGCTTCATCGAGAC	60,5°C<br>58,4°C (salt)	**Alg2_R**	AGACACCTGGTCGCACAC	62,4°C<br>58,4°C (salt)	186	103,4	0,98
15	**Mlec**	ENSMUST00000112121	**Mlec_F**	TGGGACAGTAGACGATGTACC	61,2°C<br>61,3°C (salt)	**Mlec_R**	GCTGTTGTCCGAGGCATA	59,9°C<br>56,1°C (salt)	172	101	0,99
16	**Hsp90b1**	ENSMUST00000020238	**Hsp90b1_F**	AAAGGACTTGCGACTCGCC	63,4°C<br>59,5°C (salt)	**Hsp90b1_R**	ATCAGCTCTGACGAACCCGA	63,6°C<br>60,5°C (salt)	96	100	0,99
17	**Brms1l**	ENSMUST00000059250	**Brms1l_F**	AGGAAGGCAATGGCTACAC	60,2°C<br>57,3°C (salt)	**Brms1l_R**	ACAGCACTTGTGGGACACTC	62,8°C<br>60,5°C (salt)	179	105	0,98
18	**Rpl4**		**Rpl4_F**	GCCGCTGGTGGTTGAAGATAA	61,3°C (salt)	**Rpl4_R**	CGTCGGTTTCTCATTTTGCCC	61,3°C (salt)	150		
19	**Gapdh**		**Gapdh_F**	CTCCCACTCTTCCACCTTCG	62,5°C (salt)	**Gapdh_R**	GCCTCTCTTGCTCAGTGTCC	62,5°C (salt)	189		
20	**Actb**		**Actb_F**	GGCTGTATTCCCCTCCATCG	62,5°C (salt)	**Actb_R**	CCAGTTGGTAACAATGCCATGT	60,3°C (salt)	154		

Supplementary Materials

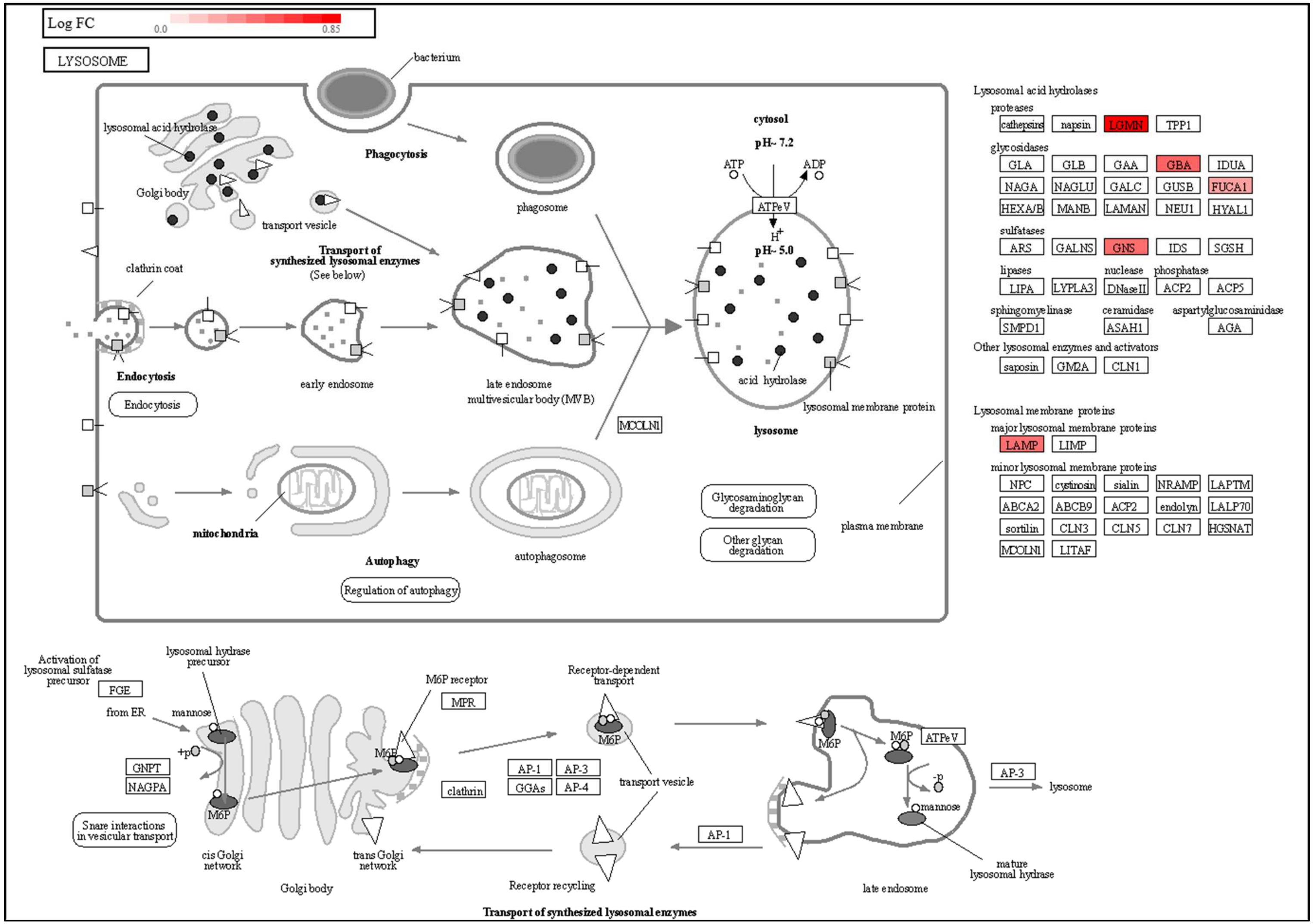

Supplementary Figure S1. Effect of Inulin on the Lysosome pathway (KEGG: 04142) from RAW 264.7 LPS-induced inflammation model cells. Pathway diagrams, overlayed with the measured protein fold-change showing coherent cascades. Differentially expressed genes are represented with positive values in red.

Supplementary Materials

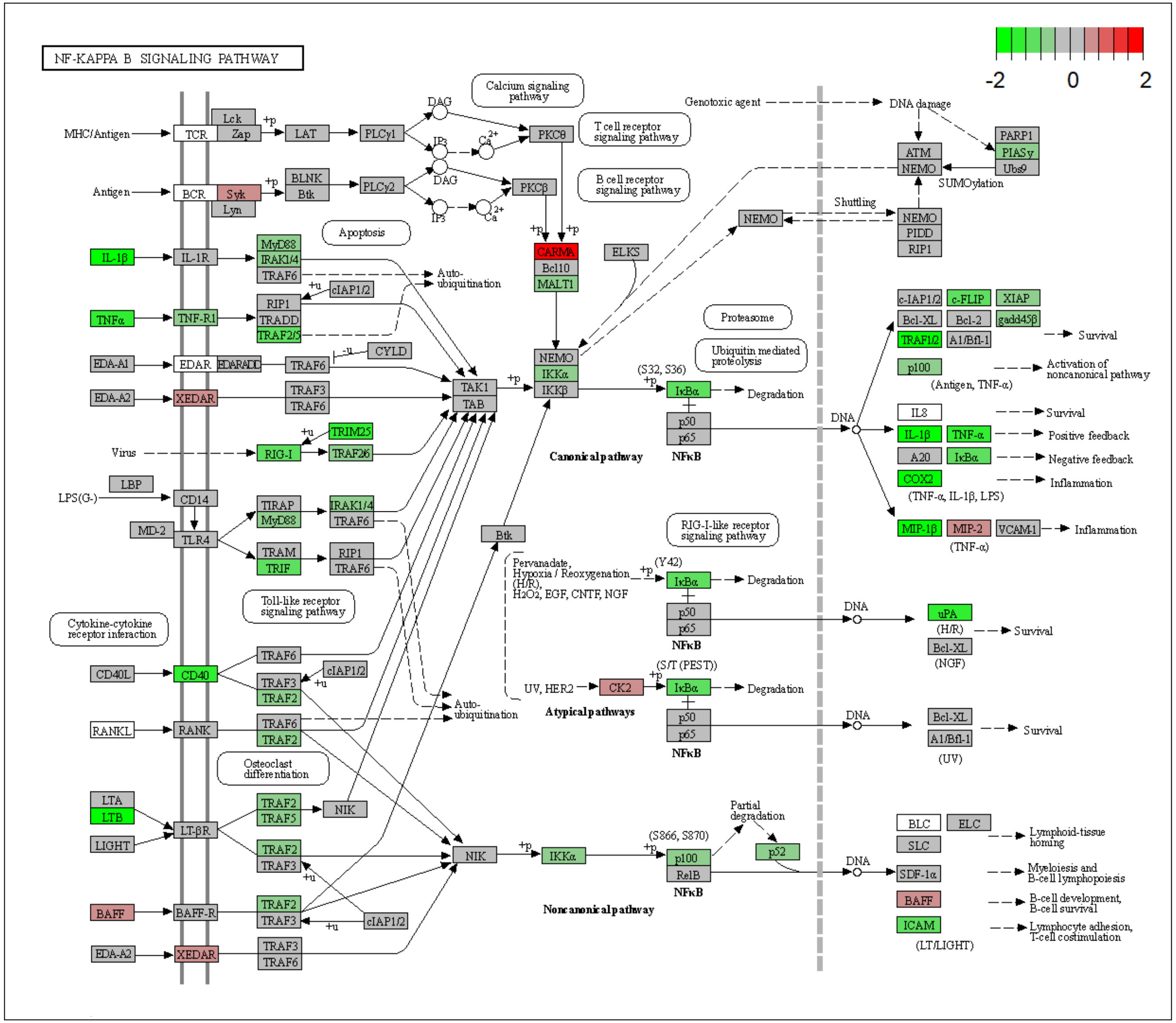

Supplementary Figure S2. Effect of inulin on the NF-κB signaling pathway showing integrated transcriptomics data. Genes over-expressed (in red) or under-expressed (green) according to the transcriptomic analysis are high-lighted.

Supplementary Materials

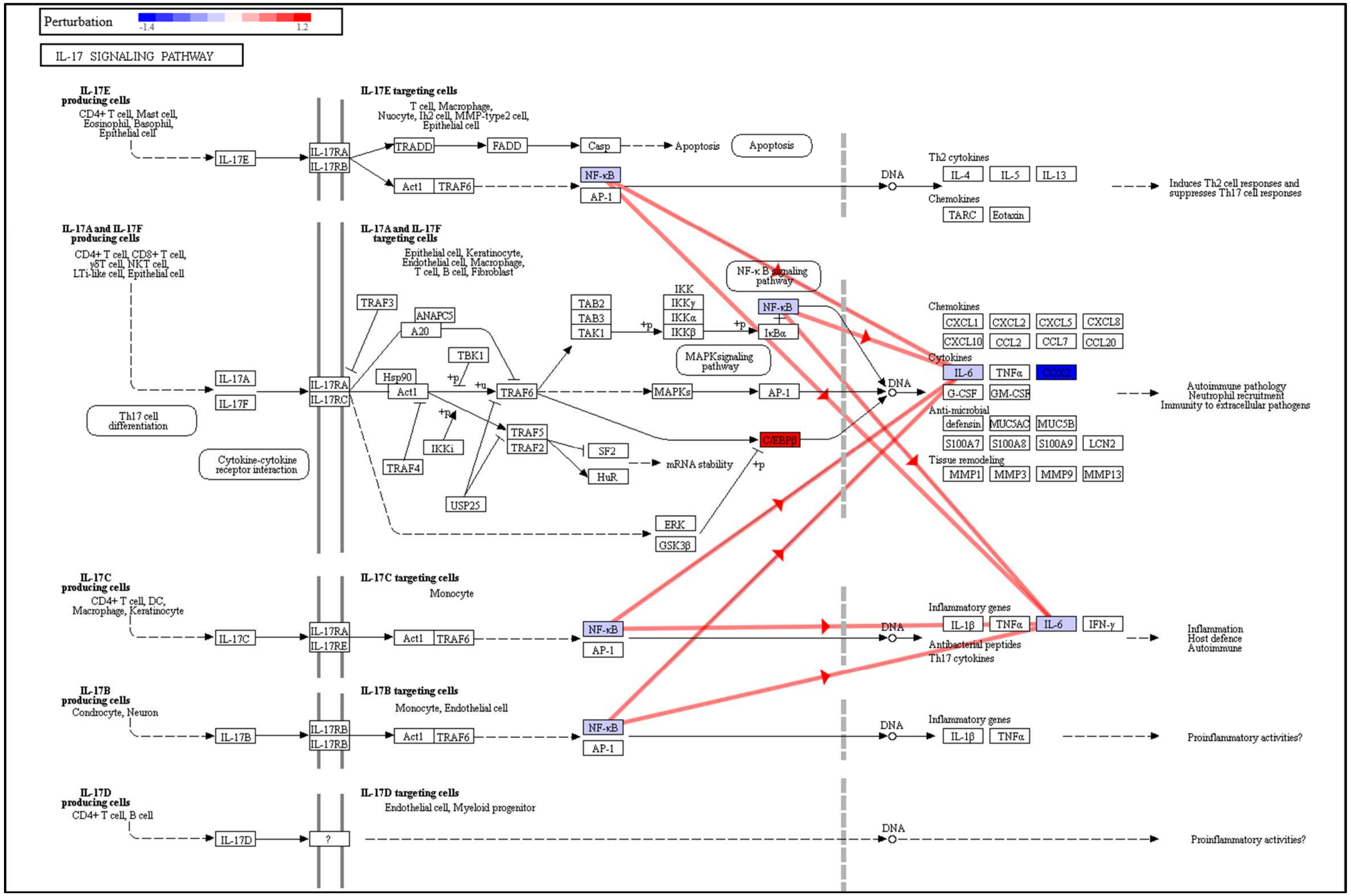

Supplementary Figure S3. Effect of Inulin on the IL-17 signaling pathway (KEGG: 04657) from RAW 264.7 LPS-induced inflammation model cells. Pathway diagrams, overlayed with the measured protein perturbation showing coherent cascades. Differentially expressed genes are represented with negative fold-change values in blue and positive values in red.
